# Retraction Note: HMGA1 exacerbates tumor growth through regulating the cell cycle and accelerates migration/invasion via targeting miR-221/222 in cervical cancer

**DOI:** 10.1038/s41419-026-08531-5

**Published:** 2026-02-27

**Authors:** Fangfang Fu, Tian Wang, Zhangying Wu, Yourong Feng, Wenwen Wang, Su Zhou, Xiangyi Ma, Shixuan Wang

**Affiliations:** 1 Department of Obstetrics and Gynecology, Tongji Hospital, Tongji Medical College, Huazhong University of Science and Technology, 430030 Wuhan, Hubei China; 2 Department of Obstetrics and Gynecology, The Affiliated Hospital of Guizhou Medical University, 55000 Guiyang, Guizhou China

Retraction Note to: *Cell Death & Disease* 10.1038/s41419-018-0683-x, published online 22 May 2018

The Editors-in-Chief has retracted the article due to concerns over image integrity

- In Figure 2, the migration and invasion panels for MS751shA1 appear highly similar.
- In Figure 5c, the 0-hour time point images for miR221 and miR222 appear highly similar.
- In Figure 6c, the panels (shcon – HMGA1) and (shA1 – cyclin D1) appear highly similar to Figure 6d panels (Sh – NC and Sh – ROR), of [1].

The Editors-in-Chief has lost confidence in the data and conclusions of this article.

Xiangyi Ma agree with this retraction and Fangfang Fu disagree with this retraction. None of the remaining authors have responded to correspondence from the Publisher about this retraction.
